# How consistent is ‘the dynamic gut’? Complex physiological responses to dietary fiber and protein across three rodent species

**DOI:** 10.1242/jeb.249797

**Published:** 2025-06-20

**Authors:** Kevin D. Kohl, Nick Barts, Karen Peralta Martínez, Anna Lackey, Emily Lyons, Matthew J. Maier, Maya Maurer, Domenique Tripoli, Tate Yawitz, Rodolfo Martínez-Mota, Bret Pasch, M. Denise Dearing, Brian K. Trevelline

**Affiliations:** ^1^ Department of Biological Sciences, University of Pittsburgh, Pittsburgh, PA 15260, USA; ^2^ Department of Biological and Clinical Sciences, University of Central Missouri, Warrensburg, MO 64093, USA; ^3^ Department of Biological Sciences, Columbia University, New York City, NY 10027, USA; ^4^ Centro de Investigaciones Tropicales, Universidad Veracruzana, Xalapa, Veracruz 91000, Mexico; ^5^ School of Biological Sciences, University of Utah, Salt Lake City, UT 84112, USA; ^6^ School of Natural Resources and the Environment, The University of Arizona, Tucson, AZ 85719, USA; ^7^ Department of Biological Sciences, Kent State University, Kent, OH 44242, USA

**Keywords:** Digestive enzymes, Integrative physiology, Nutritional ecology, Phenotypic flexibility

## Abstract

To efficiently digest food resources that may vary spatially and temporally, animals maintain physiological flexibility across levels of organization. For example, in response to dietary shifts, animals may exhibit changes in the expression of digestive enzymes, the size of digestive organs or the structure of their gut microbiome. A ‘Grand Challenge’ in comparative physiology is to understand how components of flexibility across organizational levels may scale to cumulatively determine organismal performance. Here, we conducted feeding trials on three rodent species with disparate feeding strategies: herbivorous montane voles (*Microtus montanus*), omnivorous white-footed mice (*Peromyscus leucopus*) and carnivorous grasshopper mice (*Onychomys torridus*). For each species, four groups of individuals were presented with diets that varied in carbohydrate, fiber and protein content. After 4–5 weeks, we measured organismal performance in the form of nutrient digestibility (dry matter, nitrogen, fiber). We also measured gut anatomy and organ size, and conducted enzyme assays on various tissues to measure activities of carbohydrases and peptidases. We found some shared physiological responses, e.g. fiber generally increased gut size across species. However, the specifics of these responses were distinct across species, suggesting different capacities for flexibility. Thus, in the context of digestion, we still lack an understanding of how flexibility across organizational levels may scale to determine whole-animal performance.

## Teaser

**Summary:** Rodent species with disparate feeding strategies exhibit distinct capacities for phenotypic flexibility in response to diet composition, highlighting the need for better integrative understanding of gastrointestinal flexibility.

## INTRODUCTION

Through the integrated activity of the digestive system, animals metabolize food items into constituent nutrients and energy, which are then absorbed and assimilated to fuel growth, activity and reproduction. Over evolutionary time, structures and functions of the digestive system have adapted to optimize the digestion of typical food items (Karasov and Douglas, 2013). For example, compared with the simple guts of carnivores, those of herbivorous animals have more capacious gastrointestinal tracts to retain plant material for greater fermentation (Stevens and Hume, 2004). However, the nutritional composition of animal diets may vary over space and time within an individual's lifetime. In response, animals may exhibit rapid changes to gastrointestinal physiology to maintain optimal digestion.

Understanding the nature of phenotypic plasticity, or the ability of organisms to exhibit different qualities or characteristics depending on their environment, and the role that it plays in ecology and evolution is a central focus of biology (Miner et al., 2005; Pigliucci et al., 2006; Turcotte and Levine, 2016). In particular, phenotypic flexibility represents the reversible changes that adult animals may exhibit in response to environmental variation (Piersma and Drent, 2003). For example, the gut is a dynamic organ that can respond rapidly and reversibly to short-term variation in nutritional composition (Starck, 1999). In birds and rodents, greater gastrointestinal flexibility has been associated with greater environmental climatic variability (Maldonado et al., 2011; Naya et al., 2008). It has also been hypothesized that the capacity for flexibility may be greater in generalists or omnivorous species compared with dietary specialists (Buddington et al., 1991; Karasov et al., 2011). Greater flexibility in activities of digestive enzymes may grant animals the ability to digest a greater variety of food items (Bozinovic et al., 2010; Maldonado et al., 2019). However, a meta-analysis across rodent species did not find omnivores to have greater flexibility in small intestine length when compared with other feeding strategies (Naya et al., 2008). Indeed, the capacity for this flexibility can vary, with some species or populations being more physiologically ‘fixed’ or ‘rigid’, and we still lack an understanding of the factors that cause this canalization. A major limitation towards this understanding is the lack of repeated studies using similar methodologies to understand the flexibility of the digestive system across multiple species. Under identical environmental and dietary conditions, closely related *Peromyscus* mouse species exhibit substantial differences in gut morphology and physiology (Yawitz et al., 2022), underscoring the value of comparative approaches for disentangling evolved differences from environmentally induced plasticity.

A further limitation in our understanding of the physiological flexibility of the digestive system is that the process of digestion is complex and integrated across many tissues and levels of biological organization. The capacity for flexibility has been investigated at the levels of gene expression (Herrera et al., 2022), activity of digestive enzymes (Wang et al., 2019), gut histology and cell structure (Leigh et al., 2018), gut size and macrostructure (Liu and Wang, 2007) in peripheral digestive organs such as the pancreas (Brzęk et al., 2013) or liver (Koutsos et al., 2001), and the cumulative impact of this on the ability of animals to digest food materials (assimilation efficiency; Liu and Wang, 2007; Maldonado et al., 2019; Wehrle et al., 2020). In general, flexibility in gastrointestinal physiology is thought to operate under mechanisms of chemical reactor theory and optimal digestion (Penry and Jumars, 1986; Sibly, 1981). For example, recalcitrant fiber reduces the energy density of food, and in response animals may increase gut length and volume to facilitate greater time and space for fermentation (Karasov et al., 2011). Alternatively, induction of digestive enzymes by their substrates will enhance digestion and absorption while optimizing biosynthetic energy and membrane space by dampening the expression of irrelevant enzymes (Karasov and Diamond, 1988; Karasov and Douglas, 2013).

Despite these wealth of studies, a ‘Grand Challenge of Comparative Physiology’ remains in terms of understanding how these biological levels integrate with one another and scale to the level of organismal performance (Mykles et al., 2010). From a chemical reactor theory, we still lack a thorough understanding of what major characteristics of the digestive system predict total performance (Marze, 2017). As initial glimpses, studies have shown that nestling songbirds exhibit parallel and correlated shifts in mRNA expression and enzyme activity in response to dietary shifts (Brun et al., 2021a,b). Further, in an omnivorous lizard, animals with greater small intestine surface area to volume ratios showed a greater capacity to digest protein (Kohl et al., 2016). However, we are only beginning to understand how the integration of digestive processes affects whole-animal performance in terms of growth rate or digestive efficiency, and in only a handful of organisms. Thus, comparative physiology represents a promising avenue towards systems biology, or the understanding of how genome sequences are translated into functional and dynamic organisms (Camacho et al., 2024; Noble, 2008; Strange, 2005).

To expand our understanding of gastrointestinal flexibility and its integration across biological levels, we conducted captive feeding trials on three species of wild rodents. While work on a limited number of species might limit the ability to draw inferences regarding forces of selection and evolution (Garland and Adolph, 1994), in-depth studies such as ours provide standardized methods and investigations of mechanisms, while also providing a rationale for larger studies (Goymann and Schwabl, 2021). Here, our three focal species vary in their natural feeding strategies: Montane voles (*Microtus montanus*; herbivorous; >90% of diet composed of plant material; Sera and Early, 2003); white-footed mice (*Peromyscus leucopus*; omnivorous; ∼30% insects, 30% seeds, 30% other vegetation; Lackey et al., 1985); and grasshopper mice (*Onychomys torridus*; almost exclusively carnivorous/insectivorous; McCarty, 1975). We presented these species with diets that varied in levels of protein, carbohydrates and fiber to test how these nutrients independently and interactively alter digestive physiology. We qualitatively compared species in their flexibility in digestive efficiency, organ mass, gut morphology, gut pH and activity of digestive enzymes, with the null hypothesis that all three species should show similar flexibility in response to dietary variation. Alternatively, differential responses to dietary manipulation could be exhibited across rodent species, which would contribute towards our understanding of the evolution of digestive flexibility across species.

## MATERIALS AND METHODS

We collected wild montane voles [*Microtus montanus* (Peale 1848)] near Timpie Springs Waterfowl Management Area, Dugway, Tooele Co., UT, USA (40.753708, −112.639903). Wild white-footed mice [*Peromyscus leucopus* (Rafinesque 1818)] were collected near Murray, Calloway Co., KY, USA (36.686582, −88.221204). Southern grasshopper mice [*Onychomys torridus* (Coues 1874)] were collected from field sites near Animas, Hidalgo Co., NM, USA (31.813436, −108.813772). Forty individuals of each species were collected using baited Sherman live traps under the following state permits: *M. montanus* – UT Division of Wildlife Resources, 1COLL5194-2; *P. leucopus* – Kentucky Dept of Fish and Wildlife, SC1911097; *O. torridus* – New Mexico Department of Game and Fish, #3562. All research protocols followed the guidelines of the American Society of Mammalogists (Sikes et al., 2016) and were approved by Institutional Animal Care and Use Committee (IACUC) protocols registered at University of Utah (16-02011 to M.D.D.), Murray State University (2018-026 to T. Derting) and Northern Arizona University (#15-014 and #16-001 to B.P.).

Animals were housed singly in captivity and randomly assigned to one of four diet treatments that varied in protein, carbohydrate and fiber content (Table 1). Importantly, energy density (kcal g^−1^) remained the same for low- and high-protein diets, though the inclusion of dietary fiber resulted in a dilution of energy density (Table 1).

**Table 1.** Nutritional composition and energy density of experimental diets

	Low protein, high fiber	Low protein, low fiber	High protein, high fiber	High protein, low fiber
Protein (%)	14.0	14.0	27.7	27.7
Carbohydrates (%)	26.4	52.6	12.4	39.2
NDF (%)	36.5	12.0	36.5	12.0
ADF (%)	14.9	3.3	15.6	4.1
Fat (%)	5.0	5.0	5.0	5.0
Energy density (kcal g^−1^)	2.1	3.1	2.1	3.1

NDF, neutral detergent fiber; ADF, acid detergent fiber. Percentages are on a by-mass basis.

Animals were maintained on experimental diets for a period of 4–5 weeks, after which they were euthanized with an overdose of isoflurane. During dissections, we weighed the liver, kidneys, heart, spleen and pancreas. The entire gastrointestinal tract was removed and placed upon a metal tray with ice underneath to keep the tissues chilled. Here, we collected the contents of each section: foregut, stomach, small intestine, cecum and large intestine. Using an Accumet micro-pH meter (Fisher Scientific, Waltham, MA, USA), we measured the pH of contents from each section. Each section was rinsed with sterile phosphate-buffered saline (PBS), blotted dried and weighed.

For the small and large intestines, we first measured total length by placing intestinal sections on a metal ruler wetted with PBS. Intestinal sections (small or large) were carefully stretched and allowed to gently and naturally recoil, and the length was recorded in millimeters. Then, sections from the midpoint of the small and large intestines were collected and placed in 10% buffered formalin for histological analyses. The remaining intestinal tissues were cut open longitudinally and opened flat on the iced metal tray. We used digital calipers to take 4–8 width measurements to estimate the outer circumference and diameter of the small and large intestines. The small intestine was then divided into three parts of roughly similar length (proximal, middle and distal intestine), and cut longitudinally with a razor blade, with half being frozen immediately, and the other half preserved in RNA-later for 24 h and then frozen.

### Digestibility

We measured digestibility of dry matter and specific nutrients by using acid-insoluble ash as an inert marker in the diet and feces (Furuichi and Takahashi, 1981). This process involved incinerating a small amount of feces overnight at 500°C. The resulting ash was then boiled in hydrochloric acid for 5 min, transferred to ashless filter paper and rinsed several times with 10 ml of water. Finally, the filter paper was incinerated overnight at 500°C to acquire acid-insoluble ash (AIA). We measured total fiber content (neutral detergent fiber, NDF) and cellulose/lignin content (acid detergent fiber, ADF) of food and feces using an Ankom fiber analyzer 200/200 (Ankom, Fairport, NY, USA). The percentage nitrogen content of fecal samples was measured by the Stable Isotope Ratio Facility ​for Environmental Research (University of Utah) using an EA 1110 elemental analyzer (CE Instruments, Wigan, UK) coupled with a DELTAplus Advantage isotope ratio mass spectrometer (Thermo Fisher Scientific, Waltham, MA, USA) to measure the percentage of nitrogen in each sample. Apparent digestibility of dry matter and nutrients (NDF, ADF, crude protein) was calculated using AIA data and digestibility balance formulas (De Marco et al., 2012).

### Histology

Intestinal sections collected from the midpoint of the small and large intestines were fixed in a 10% buffered formalin solution. Tissue samples were then dehydrated through a graded series of 70%, 96% and 100% ethanol solution, then clarified two times in xylene, and embedded in paraffin at 56°C for 2 h. We obtained cross-sections using a universal rotary microtome. Samples were then mounted on slides, stained with hematoxylin and eosin, and covered with cover glasses. Microphotographs were taken using an Olympus BX50 microscope connected to a video camera (HDCE-30C) and a PC-based image analysis system using Image J software (Schneider et al., 2012). For the small intestine, we measured muscle layer thickness, villus height, villus width, crypt depth and crypt width. For the large intestine, we measured muscle layer thickness, mucosa thickness, crypt depth and crypt width. For all measurements, we acquired at least 4–10 replicate measures per individual, which were then averaged within an individual. The histological sections of some individuals were not of sufficient structure to collect all measurements (especially small intestine villi). Full data are available in Dataset 1.

### Enzyme assays

The activity of pancreatic amylase was measured by a modification of the 3,5-dinitrosalicylate method (Dahlqvist, 1962). Several pieces of pancreas were thawed and homogenized for 30 s using 10 ml g^−1^ tissue of 50 mmol l^−1^ Tris/HCl buffer [pH 6.9, containing 3 mmol l^−1^ taurocholic acid, 0.27% (w/v) Triton X-100, 1 mmol l^−1^ benzamidine and 2 mmol l^−1^ hydrocinnamic acid]. Homogenate aliquots of 100 μl were incubated with 100 μl of 2% potato starch (Sigma S2630) at 37°C for 3 min. The reaction was terminated by the addition of 200 μl dinitrosalicylate reagent. Blank samples contained exactly the same ingredients, but dinitrosalicylate was added before the substrate and then handled in the same way as other samples. The tubes were immersed in boiling water for 10 min and cooled with tap water. Then, 1 ml distilled water was added to each tube, the absorbance was read at 530 nm, and enzyme activity was calculated using a glucose standard curve.

We followed establish protocols for the measurement of pancreatic trypsin (Erlanger et al., 1961). Here, several pieces of pancreatic tissue were homogenized for 30 s using 10 ml g^−1^ tissue of 50 mmol l^−1^ Tris/HCl buffer [pH 8.2, containing 3 mmol l^−1^ taurocholic acid and 0.27% (w/v) Triton X-100]. To activate zymogens, 16 μl of the homogenate samples was incubated with 0.3% enterokinase for 20 min at 37°C (Sigma E0632 in 50 mmol l^−1^ Tris/HCl buffer pH 8.2 containing 20 mmol l^−1^ CaCl_2_), and centrifuged for 10 min at 20,000 ***g*** at 4°C to remove a white suspension that sometimes appears in solution. This activated supernatant (16 μl) was then incubated with 144 μl distilled water and 800 μl of 1 mmol l^−1^ DL-BAPNA (benzoyl-arginine-*p*-nitroanilide; pH 8.2) solution as the substrate for 10 min at 37°C. The reaction was terminated by adding 160 μl of 30% acetic acid. Blank samples contained exactly the same ingredients, but acetic acid was added before the substrate and then handled in the same way as other samples. The liberated amount of *p*-nitroaniline was estimated by reading the absorbance at 410 nm.

For intestinal enzymes, tissue samples from the middle section of the small intestine were thawed over ice and immediately homogenized with cold homogenizing buffer (300 mmol l^−1^ mannitol in 1 mmol l^−1^ Hepes/KOH, pH 7; Sigma-Aldrich Corp., St Louis, MO. USA, CAS number 7365-45-9) with an OMNI TH-01 tissue homogenizer (Omni International, Kennesaw, GA, USA) set at ∼20,000 rpm for 30 s. For disaccharidase activity (maltase and sucrase), we diluted tissue homogenates in the homogenizing buffer above (maltase: 2 μl of homogenate in 498 μl of homogenizing buffer; sucrase: 10 μl of homogenate in 90 μl of homogenizing buffer). Then, we took 30 μl of the diluted tissue homogenate and incubated it for 20 min at 37°C in a 1.5 ml vial containing 30 μl of maleate/NaOH buffer (0.1 maleate/1 mol l^−1^ NaOH, pH 7) containing 56 mmol l^−1^ maltase or sucrase, depending on the enzyme of interest. Each assay was performed in triplicate. The reactions were stopped with the addition of 400 µl of Glucose-Assay-Kit stop solution (GAGO-20 glucose assay kit; Sigma Aldrich) to each vial. All the replicates were incubated at 37°C for 30 min, after which 400 μl of 6 mol l^−1^ H_2_SO_4_ was added to each tube, and the absorbances were measured at 540 nm using a plate reader (BioTek Synergy H1, Winooski, VT, USA). Using a glucose standard curve, the activity of maltase and sucrase was calculated as the average of triplicate absorbance measurements for each sample minus blanks and expressed as µmol min^−1^ g^−1^ wet tissue.

Aminopeptidase-N activity was assayed using l-alanine-*p*-nitroanilide (Sigma-Aldrich, item # A9325) as a substrate (Brzęk et al., 2013). Each reaction began in a 1.5 ml vial upon mixing 2.5 μl of tissue homogenate with 250 µl of assay solution [0.1 mol l^−1^ phosphate buffer (NaH_2_PO_4_/Na_2_HPO_4_, pH 7.0) containing 2.0 mmol l^−1^ l-alanine-*p*-nitroanilide]. After 20 min of incubation at 37°C, each reaction was stopped by adding 250 µl of chilled 2 mol l^−1^ acetic acid to the vial. Absorbance was measured at 380 nm using a plate reader (as above). After subtracting background absorbance, we used a calibration standard curve made with dilutions of l-alanine-*p*-nitroanilide to calculate aminopeptidase-N activity in units of µmol min^−1^ g^−1^ wet tissue.

### Statistical analyses

Given the substantial differences in body size and physiology across our species, we largely analyzed our data separately for each species and qualitatively compared across species. We used ANOVA to compare measurements of body mass, food intake, digestibility, luminal pH values and activity of digestive enzymes. Data for measures of fiber digestibility (both NDF and ADF digestibility) did not follow a normal distribution, and thus we used a generalized linear model with a Poisson distribution to compare effects of diet on these variables. Organ masses and morphometrics were analyzed with the inclusion of body mass as a covariate (resulting in ANCOVA, with data being checked for normality of residuals). Graphically, we present residuals of organ mass and morphometrics from regressions with body mass.

To compare the integration of flexibility across species, we conducted independent principal component analyses (PCA) to the correlation matrices of the measurements listed above, using residuals for organ mass, and absolute values for luminal pH and enzyme activity. Because of sparse data availability, we did not include histological measurements, or foregut, stomach or large intestine pH values (the small intestine and cecal pH were included). We then compared principal component (PC) values using multifactor ANOVA across all samples, and comparisons of dietary treatments within a species. All statistical tests were performed using JMP 17, with statistical significance defined as *P*<0.05.

## RESULTS

### Whole-animal metrics (body mass, digestibility)

**Fig. 1.**
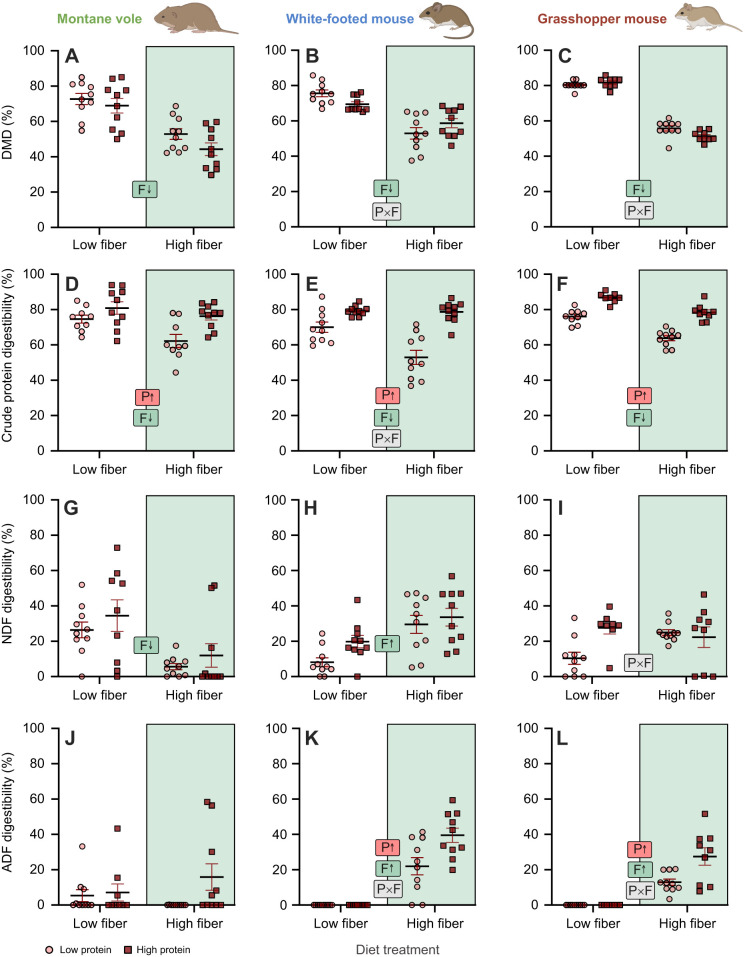
**Effects of diet composition on nutrient digestibility at the whole-animal level for the three rodent species.** (A–C) Dry matter digestibility, (D–F) crude protein digestibility, (G–I) neutral detergent fiber (NDF) digestibility and (J–L) acid detergent fiber (ADF) digestibility in montane voles (left), white-footed mice (middle) and grasshopper mice (right) fed one of four diets that varied in protein, carbohydrate and fiber content. Letters and arrows inset within panels summarize significant experimental effects based on statistical results presented in Table 2: P, protein effect; F, fiber effect; P×F, protein×fiber effect. Rodent images were created in BioRender by Kohl, K., 2025. https://BioRender.com/454aukj. This figure was sublicensed under CC-BY 4.0 terms.

Following 4–5 weeks of exposure to the different diets, groups did not differ significantly in body mass as an effect of protein, fiber or any interaction for any of the rodent species. We found that diet macronutrient composition significantly impacted the ability of rodents to digest various components of their diets, and that these patterns varied across species, suggesting differential responses of the overall digestive system. In montane voles, we found that dry matter digestibility was significantly reduced by ∼22% when animals were fed diets containing high fiber (Fig. 1A, Table 2), with no significant interaction between dietary protein and fiber. In white-footed mice, high fiber also significantly decreased dry-matter digestibility, by ∼17%. We also observed a significant interaction between dietary fiber and protein, though differences across groups based on *post hoc* comparisons were only significant based on dietary fiber levels (Fig. 1B, Table 2). Similarly, in grasshopper mice, we observed significant effects of fiber, with fiber decreasing dry-matter digestibility by ∼22%. Here, we also observed a significant interaction between dietary protein and fiber, such that when feeding on diet containing high fiber, increased protein levels resulted in grasshopper mice exhibiting lower dry-matter digestibility (Fig. 1C, Table 2).

**Table 2.** Statistical results of ANOVA for digestive performance

	Montane vole			White-footed mouse			Grasshopper mouse		
Digestibility	*F*	χ^2^	*P*	*F*	χ^2^	*P*	*F*	χ^2^	*P*
Dry matter									
Fiber	39.31	** **	**<0.0001**	45.11	** **	**<0.0001**	597.46	** **	**<0.0001**
Protein	3.58		0.068	0.02		0.89	2.03		0.16
Fiber×Protein	0.60		0.44	6.22	** **	**0.018**	7.04	** **	**0.012**
Protein									
Fiber	7.40	** **	**0.011**	9.78	** **	**0.004**	50.44	** **	**<0.0001**
Protein	10.54	** **	**0.003**	40.54	** **	**<0.0001**	82.34	** **	**<0.0001**
Fiber×Protein	1.41		0.24	9.46	** **	**0.004**	2.39		0.13
NDF									
Fiber		25.65	**<0.0001**		27.38	**<0.0001**		23.18	**0.003**
Protein		246.47	**<0.0001**		139.88	**<0.0001**		8.83	**<0.0001**
Fiber×Protein		0.36	0.545		6.27	0.0123		42.67	**<0.0001**
ADF									
Fiber		143.26	**<0.0001**		802.19	**<0.0001**		464.71	**<0.0001**
Protein		32.58	**<0.0001**		<0.01	0.99		<0.01	0.99
Fiber×Protein		100.58	**<0.0001**		<0.01	0.99		<0.01	0.99

For dry matter and protein digestibility, degrees of freedom (model, error) for each species are as follows: voles (3,34); white-footed mice (3,36); and grasshopper mice (3,32). Digestibility of NDF and ADF was compared using a generalized linear model using the Poisson distribution. Bold indicates significance.

We observed more consistent responses across species in how dietary composition impacted the digestibility of crude protein. For all three species, the efficiency of protein digestion was higher when animals were consuming high-protein diets (Fig. 1D–F, Table 2). Dietary fiber also had the effect of lowering protein digestibility in all three rodent species. In white-footed mice, we observed a significant interaction between dietary protein and fiber, such that protein digestibility was lowest on a low-protein high-fiber diet (Fig. 1E, Table 2).

Rodent species exhibited differential responses in how diet composition impacted their ability to digest total fiber content (NDF) and cellulose/lignin content (ADF). In montane voles, individuals consuming high-fiber diets exhibited lower efficiency of digesting NDF. Conversely, in white-footed mice, the efficiency of digesting NDF was higher for animals feeding on high-fiber diets. Grasshopper mice exhibited a significant fiber×protein interaction, such that digestibility of NDF was lowest on the low-protein, low-fiber diet (Fig. 1I, Table 2). For the digestion of fiber, montane voles exhibited a complex interaction in how nutrients affected their ability to digest ADF, or the lignin and cellulose portion of the diet (Fig. 1J, Table 2). In both white-footed mice and grasshopper mice, we found that positive ADF digestibility was only detectable when animals were being fed high-fiber diets (Fig. 1K,L, Table 2).

### Gastrointestinal mass

**Fig. 2.**
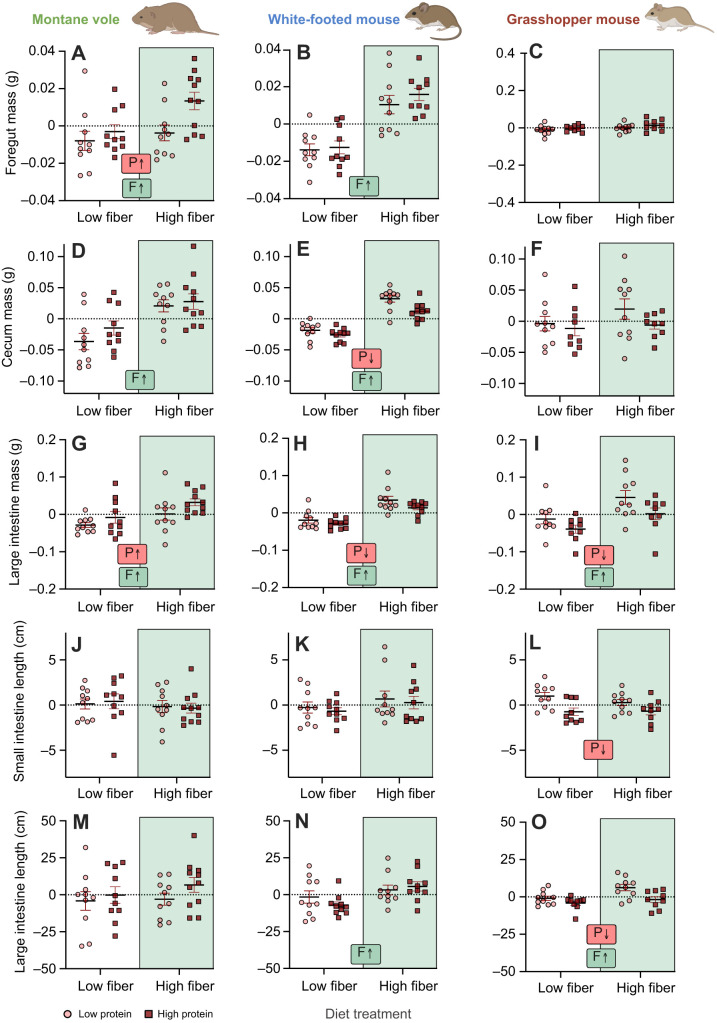
**Effects of diet composition on foregut, cecum and large intestine mass and intestinal length of the three rodent species.** (A–C) Foregut mass, (D–F) cecum mass, (G–I) large intestine mass, (J–L) small intestine length and (M–O) large intestine length of montane voles (left), white-footed mice (middle) and grasshopper mice (right) fed one of four diets. Graphically, points represent residuals of organ mass from correlations with body mass. Statistically, models were run as multifactor ANCOVA using body mass as a covariate. Letters and arrows inset within panels summarize significant experimental effects based on statistical results presented in Table 3: P, protein effect; F, fiber effect. Rodent images were created in BioRender by Kohl, K., 2025. https://BioRender.com/454aukj. This figure was sublicensed under CC-BY 4.0 terms.

The effect of diet on the mass of gastrointestinal regions varied considerably across species, with distinct responses to dietary components. Both dietary fiber and protein resulted in an increase in the mass of the foregut region in montane voles, while the foregut mass of white-footed mice was only influenced by dietary fiber, and the grasshopper mice showed no change in foregut mass (Fig. 2, Table 3). The cecal chamber also showed distinct responses, with white-footed mice being the only species exhibiting smaller cecal chambers on high-protein diets. In regard to the effects of fiber, white-footed mice had 61% larger cecal chambers when feeding on high-fiber diets, while montane voles only increased their cecal mass by 16%, and grasshopper mice did not exhibit significant differences in cecum mass (Fig. 2, Table 3). The effects of protein were distinct, such that montane voles showed greater large intestine mass on high-protein diets, while white-footed mice and grasshopper mice both exhibited lower large intestine mass on high-protein diets (Fig. 2, Table 3). Dietary fiber had more consistent results on the mass of the large intestine, where fiber increased the mass of this tissue by 11%, 47% and 24% in montane voles, white-footed mice and grasshopper mice, respectively (Fig. 2, Table 3). Data for stomach and small intestine mass can be found in Fig. S1.

**Table 3.** Statistical results of ANCOVA for digestive organ mass and gastrointestinal morphometrics, using body mass as a covariate

	Montane vole		White-footed mouse		Grasshopper mouse	
	*F*	*P*	*F*	*P*	*F*	*P*
Foregut mass						
Fiber	5.01	**0.03**	50.67	**<0.0001**	3.39	0.07
Protein	6.09	**0.02**	0.54	0.47	2.07	0.16
Fiber×Protein	1.93	0.17	0.38	0.54	0.14	0.71
Body mass	14.09	**0.0006**	24.55	**<0.0001**	10.31	**0.003**
Cecum mass						
Fiber	17.43	**0.0002**	115.32	**<0.0001**	1.47	0.23
Protein	1.37	0.25	12.48	**0.001**	1.85	0.18
Fiber×Protein	0.39	0.53	1.98	0.16	0.51	0.48
Body mass	19.18	**<0.0001**	30.90	**<0.0001**	1.39	0.25
Large intestine mass						
Fiber	7.66	**0.009**	47.43	**<0.0001**	11.99	**0.002**
Protein	4.32	**0.045**	5.06	**0.03**	5.97	**0.02**
Fiber×Protein	0.20	0.66	0.50	0.48	0.32	0.57
Body mass	26.11	**<0.0001**	12.15	**0.0013**	4.66	**0**.**038**
Small intestine length		** **		** **		
Fiber	0.62	0.44	2.06	0.16	0.59	0.45
Protein	0.01	0.93	0.40	0.53	10.85	**0.002**
Fiber×Protein	0.12	0.72	0.001	0.99	0.93	0.34
Body mass	13.26	**0.0008**	2.15	0.15	47.16	**<0.0001**
Small intestine diameter		** **		** **		** **
Fiber	0.48	0.49	2.39	0.13	4.14	**0.05**
Protein	0.12	0.73	5.50	**0.02**	1.73	0.20
Fiber×Protein	0.10	0.75	0.18	0.67	2.47	0.13
Body mass	21.59	**<0.0001**	1.07	0.31	1.90	0.18
Large intestine length		** **		** **		
Fiber	0.53	0.47	7.43	**0.01**	7.53	**0.009**
Protein	1.67	0.20	0.31	0.58	10.04	**0.003**
Fiber×Protein	0.30	0.58	1.53	0.22	1.81	0.19
Body mass	0.24	0.63	2.85	0.10	3.28	0.08
Large intestine diameter						
Fiber	5.07	**0.03**	19.29	**<0.0001**	4.35	**0.04**
Protein	2.17	0.15	0.09	0.77	0.93	0.34
Fiber×Protein	3.25	0.08	2.98	0.09	0.001	0.98
Body mass	1.08	0.30	0.02	0.87	0.04	0.83

Degrees of freedom (model, error) for each species are as follows: voles (4,36); white-footed mice (4,35); and grasshopper mice (4,33). Bold indicates significance.

### Intestinal morphometrics

At the level of gross morphology, we also observed variable responses to diet across the three rodent species. Grasshopper mice exhibited significantly longer small intestines when feeding on low-protein diets (Fig. 2, Table 3), while white-footed mice exhibited significantly wider small intestines (in terms of diameter) when feeding on low-protein diets (Fig. S2, Table S1). Dietary fiber also increased the small intestine diameter of grasshopper mice (Fig. S2, Table S1).

Responses of large intestinal morphology were somewhat more consistent across species compared with those of the small intestine. Here, dietary fiber increased large intestine length in both white-footed mice and grasshopper mice, and increased large intestine diameter in all three rodent species (Fig. S2, Table S1). We also observed that grasshopper mice feeding on low-protein diets exhibited longer large intestines (Fig. 2, Table 3).

At a histological level, we observed few differences based on diet. We found only a significant fiber×protein interaction for villus width in montane voles, but not for other rodent species (Fig. S2). We did not observe significant differences in muscle thickness, villus height, crypt depth or crypt width in the small intestine, nor any differences in muscle thickness, mucosa thickness, crypt depth or crypt width in the large intestine (see Dataset 1).

### Peripheral organ mass

We observed variable responses across rodent species in how the mass of peripheral organs differed according to diet composition. In all three species, protein significantly increased the mass of the pancreas (Fig. S1, Table S1). In montane voles and white-footed mice, fiber also independently increased the mass of the pancreas. Kidney mass of all three species was greater in animals consuming high-fiber diets (Fig. S1, Table S1). In grasshopper mice, we observed that fiber also independently increased kidney mass. Finally, the mass of the spleen was responsive to diet in montane voles, which also exhibited far greater variation in spleen size compared with the other two species (Fig. S1, Table S1). Both dietary fiber and protein independently yielded greater spleen mass for montane voles, but not other rodent species. Liver mass did not vary as an effect of diet in any species (Dataset 1).

### Luminal pH

**Fig. 3.**
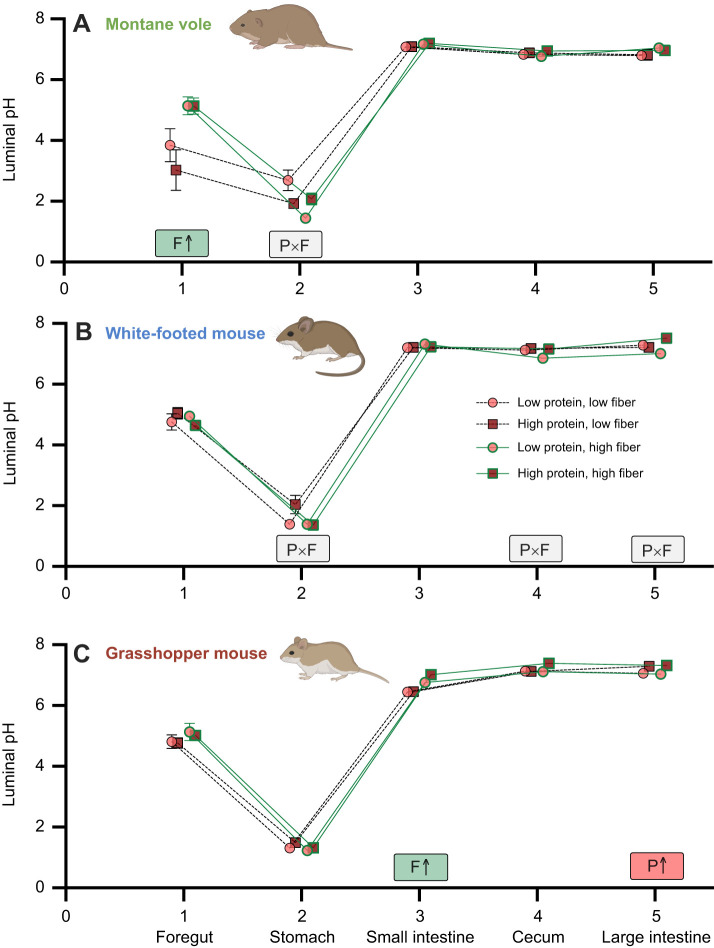
**Effects of diet composition on the gastrointestinal pH of various gut regions of the three rodent species.** Luminal pH of (A) montane voles, (B) white-footed mice and (C) grasshopper mice. Letters and arrows inset within panels summarize significant experimental effects based on statistical results presented in Table 4: P, protein effect; F, fiber effect; P×F, protein×fiber effect. Rodent images were created in BioRender by Kohl, K., 2025. https://BioRender.com/454aukj. This figure was sublicensed under CC-BY 4.0 terms.

Rodent species exhibited differential responses in gastrointestinal pH to varying diet composition. We found that montane voles fed high-fiber diets maintained foregut chambers with significantly higher pH, while stomach pH of montane voles depended on an interaction between protein and fiber levels (Fig. 3A, Table 4). In white-footed mice, luminal pH of the stomach, cecum and large intestine varied depending on interactions between dietary protein and fiber (Fig. 3B, Table 4). Lastly, in grasshopper mice, animals fed high-fiber diets exhibited higher small intestine pH, and animals fed diets containing high-protein exhibited higher large intestine pH (Fig. 3C, Table 4).

**Table 4.** Statistical results of ANOVA for luminal pH of gastrointestinal regions

	Montane vole			White-footed mouse			Grasshopper mouse		
	d.f.	*F*	*P*	d.f.	*F*	*P*	d.f.	*F*	*P*
Foregut pH									
Fiber	3,33	14.43	**0.0006**	3,33	0.34	0.57	3,29	1.66	0.21
Protein		0.84	0.36		0.00	0.97		0.14	0.71
Fiber×Protein		0.80	0.38		2.53	0.12		0.02	0.88
Stomach pH									
Fiber	3,36	7.13	**0.01**	3,35	4.01	**0.05**	3,34	1.56	0.22
Protein		0.09	0.76		3.25	0.08		1.74	0.19
Fiber×Protein		11.40	**0.002**		3.86	**0.05**		0.17	0.68
Small intestine pH									
Fiber	3,37	2.96	0.09	3,36	0.46	0.50	3,34	14.28	**0.0006**
Protein		0.04	0.83		0.12	0.73		1.41	0.24
Fiber×Protein		0.01	0.91		0.31	0.58		1.11	0.30
Cecal pH									
Fiber	3,37	0.00	0.97	3,36	5.41	**0.03**	3,32	1.14	0.29
Protein		2.86	0.09		9.09	**0.005**		1.27	0.27
Fiber×Protein		0.86	0.36		4.44	**0.04**		1.46	0.24
Large intestine pH					** **	** **			** **
Fiber	3,37	1.97	0.17	3,34	0.02	0.90	3,25	0.00	0.99
Protein		0.02	0.88		4.86	**0.03**		4.89	**0.03**
Fiber×Protein		0.14	0.70		8.94	**0.005**		0.04	0.83
Pancreatic trypsin						** **			
Fiber	3,35	0.85	0.36	3,36	0.06	0.81	3,33	5.64	**0.02**
Protein		0.01	0.95		4.89	**0.03**		10.41	**0.003**
Fiber×Protein		0.07	0.79		1.37	0.25		4.84	**0.035**
Pancreatic amylase^a^						** **			
Fiber	3,35	3.61	0.07	3,36	8.01	**0.007**	3,33	0.35	0.56
Protein		0.13	0.71		1.98	0.17		16.81	**0.0003**
Fiber×Protein		0.44	0.51		0.08	0.77		0.06	0.81
Intestinal maltase^a^						** **			
Fiber	3,35	54.29	**<0.0001**	3,36	16.45	**0.0003**	3,33	4.63	**0.039**
Protein		6.16	**0.018**		6.11	**0.018**		5.41	**0.026**
Fiber×Protein		4.49	**0.041**		1.38	0.25		1.43	0.24

^a^The activities of these carbohydrase enzymes may be better explained by carbohydrate content in the diet. See Table 1. Bold indicates significance.

### Digestive enzyme activity

**Fig. 4.**
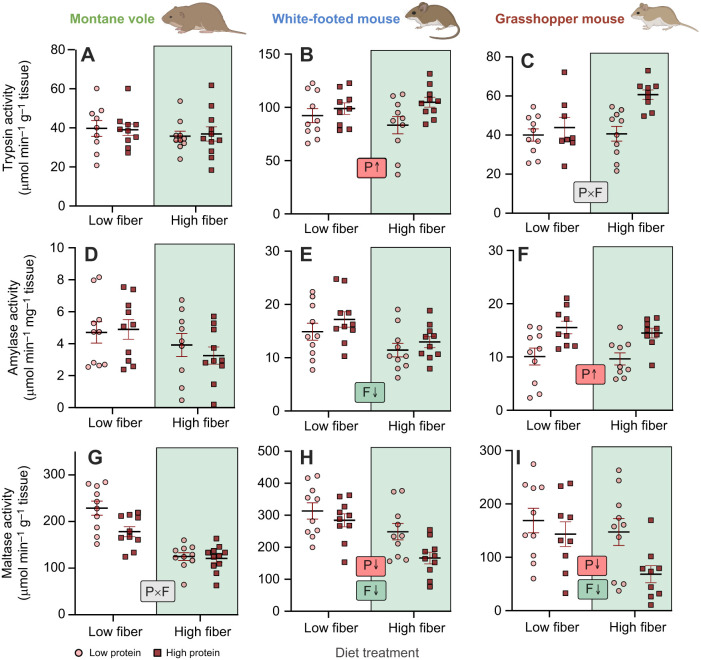
**Effects of diet composition on digestive enzyme activity in the three rodent species.** (A–C) Trypsin activity, (D–F) amylase activity and (G–I) maltase activity of montane voles (left), white-footed mice (middle) and grasshopper mice (right) fed one of four diets. Letters and arrows inset within panels summarize significant experimental effects based on statistical results presented in Table 4: P, protein effect; F, fiber effect; P×F, protein×fiber effect. Note, diets also vary in carbohydrate content, and are organized in order from highest carbohydrate content (low-fiber, low-protein: 52.6%) to lowest carbohydrate content (high-fiber, high-protein: 12.4%). Rodent images were created in BioRender by Kohl, K., 2025. https://BioRender.com/454aukj. This figure was sublicensed under CC-BY 4.0 terms.

We also observed differential responses in the activity of digestive enzymes to variation in diet. In white-footed mice, animals feeding on high-protein diets had higher mass-specific activity of the pancreatic protease trypsin (Fig. 4, Table 4). Similar responses were observed in grasshopper mice, though the magnitude of the response was larger in the context of a high-fiber diet (i.e. a significant protein×fiber interaction). Montane voles did not exhibit any flexibility in pancreatic trypsin activity (Fig. 4, Table 4). We did not observe any significant effects of diet on the activity of the intestinal protease aminopeptidase-N in any species. Carbohydrate-active enzymes exhibited some changes in response to diet (Fig. 4, Table 4), though the activities of these enzymes are seemingly better explained by the carbohydrate content of our experimental diets (Table 1).

### Integration

**Fig. 5.**
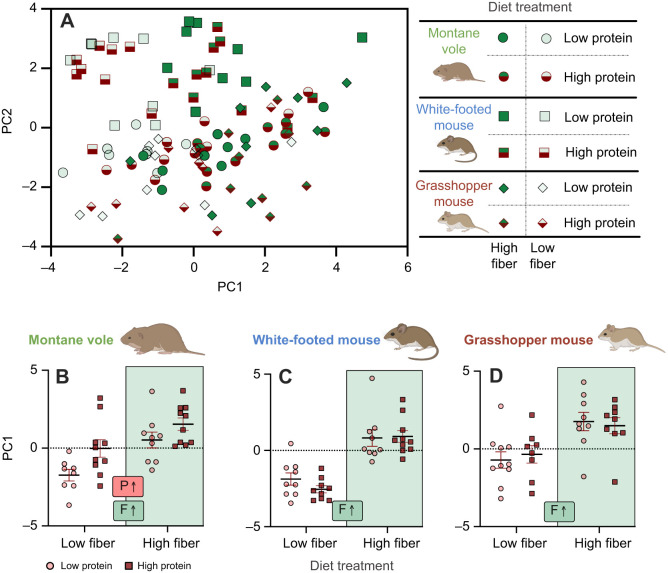
**Integrative responses of digestive morphology and physiology to nutritional composition.** (A) Principal component (PC) 1 primarily consisted of gut anatomical structures, while PC2 consisted of digestive enzyme activity and small intestine pH. (B–D) Results of ANOVA on the differences in PC1 for montane voles (B), white-footed mice (C) and grasshopper mice (D). Letters and arrows inset within panels summarize significant experimental effects based on statistical results presented in Table 5: P, protein effect; F, fiber effect. Rodent images were created in BioRender by Kohl, K., 2025. https://BioRender.com/454aukj. This figure was sublicensed under CC-BY 4.0 terms.

Finally, we compared the integration of these responses across rodent species through the use of PCA (Fig. 5A). Here, the factors that primarily loaded on the first principal component consisted of gut anatomical structures, while the factors loading on component 2 were activity of several digestive enzymes and small intestine pH (Table 5). When conducting a multi-factor ANOVA on PC1 values for all individuals, dietary fiber treatment had the largest and most significant effect (*F*=73.34; *P*<0.0001), with rodent species having a lesser effect (*F*=6.52; *P*=0.002). In the analyses of all PC1 values, we also observed a significant species×protein effect (*F*=3.33; *P*=0.039), demonstrating that species respond differently to this nutrient. Principal component 2 did not show any significant differences across variables tested.

**Table 5.** Statistical details of principal component analysis on organ mass, morphometrics, small intestine pH and digestive enzyme activity

	Principal component 1		Principal component 2	
	Factor	Loading	Factor	Loading
	Large intestine mass	0.776	Aminopeptidase-N activity	0.827
	Stomach mass	0.724	Maltase activity	0.704
	Foregut mass	0.692	Trypsin activity	0.690
	Cecum mass	0.653	Sucrase activity	0.686
	Large intestine length	0.555	Small intestine luminal pH	0.519
Eigenvalue	3.86		3.20	
Variance explained	19.3%		16%	

To better understand the differences in PC1, we conducted ANOVA on these values for each species separately (Fig. 5B–D). Here, we observed that fiber had significant effects on PC1 for all three species, though effect sizes were considerably greater in white-footed mice (fiber effects: montane voles: *F*=15.81, *P*=0.0004; white-footed mice: *F*=57.33, *P*<0.0001; grasshopper mice: *F*=15.31, *P*=0.0004). We also observed that protein had a significant effect on the PC1 values in herbivorous montane voles (*F*=8.11, *P*=0.008) but not in other rodent species (*P*>0.05), underlying the significant species×protein interaction mentioned above. We did not observe significant interactions between dietary fiber and protein.

## DISCUSSION

Here, we conducted a feeding trial on three species of rodents to test whether physiological responses were consistent or varied, and how these changes might integrate to determine whole-animal performance at the level of digestibility. Overall, we found that our three rodents exhibited differences in whole-animal performance metrics (digestive efficiency) and markedly different responses to diet across multiple tissues and measurements, including gut pH, gastrointestinal and peripheral organ masses and digestive enzyme activities. Below, we discuss these changes in relation to our current understanding of physiological mechanisms for flexibility in these traits. The difference in capacity for flexibility may be related to the natural feeding ecology and evolutionary history of each species. The omnivorous white-footed mouse exhibited greater flexibility than the herbivorous montane vole or insectivorous grasshopper mouse. Limited flexibility may be due to physiological canalization, with montane voles exhibiting constitutive expression of a higher gut capacity, whereas grasshopper mice may have lost the physiological responses to high-fiber diets. We discuss some of our findings in the context of these evolutionary hypotheses, though these conclusions are based on single-species representatives from each feeding category, and so conclusions regarding generality across feeding strategies require further testing (Garland and Adolph, 1994). However, studies such as ours with consistent methodologies provide deeper insight into mechanisms and a rationale for larger phylogenetic studies (Goymann and Schwabl, 2021).

Some effects of diet on the efficiency of digestion were shared across our rodents and previous studies. Across all three species, we saw that high-fiber diets decreased dry-matter digestibility, which is common in other studies, as fiber material moves through the gastrointestinal tract undigested and is defecated (Bozinovic et al., 1997; Hume et al., 1993; Pei et al., 2001; van Zyl et al., 1999). While not measured in our study, it is quite likely that animals increased food consumption on high-fiber diets to maintain energy assimilation, as has been shown in other rodent species (Hume et al., 1993). Moreover, this response of greater food intake is likely, given that all rodents maintained consistent body mass in the face of lower digestibility of an energy-dilute diet. Across all three species, we also saw greater efficiency of protein digestion when animals were consuming high-protein diets, suggesting acclimation of the digestive system to digest and absorb dietary protein. Across all three species, we also observed that dietary fiber decreased the efficiency of protein digestion. A reduction in protein digestibility due to fiber has also been observed in feeding trials with Mongolian gerbils (*Meriones unguiculatus*; Pei et al., 2001). However, herbivorous degus (*Octodon degus*) maintain protein digestion in the face of high-fiber diets (Bozinovic et al., 1997).

Rodents showed idiosyncratic responses in how diet composition impacted their efficiency of fiber digestion. Herbivorous voles exhibited decreases in the efficiency of fiber digestion when consuming high-fiber diets, meaning more consumed fiber moved through the gut undigested. This result is in agreement with predictive computational models of fiber utilization by small herbivores, especially given the limited ability of small-bodied herbivores to conduct effective fermentation (Justice and Smith, 1992). Empirically, granivorous Mongolian gerbils (*M. unguiculatus*; Pei et al., 2001) exhibit a lower efficiency of digesting fiber when consuming high-fiber diets, whereas herbivorous degus maintain efficiency of fiber digestion when fed increasing levels of dietary fiber (Bozinovic, 1995). In our trial, herbivorous voles exhibited decreases in the efficiency of fiber digestion, while omnivorous white-footed mice exhibited increases in the efficiency of fiber digestion when feeding on high-fiber diets. One possible explanation for the differences across studies is the use of different compounds as experimental fiber. Most previous diet trials employed powdered cellulose as a dietary fiber (Bozinovic et al., 1997; Hume et al., 1993; Pei et al., 2001; van Zyl et al., 1999). However, recent biomedical studies in humans have demonstrated that complex bran fibers elicit different microbial and physiological effects compared with powdered cellulose (Deehan et al., 2022). In our study, we used complex fiber sources in the form of oat hulls and wheat middlings. Historically, fiber has been treated as a bulk nutritional category, but moving forward it is necessary to better understand the molecular diversity of fibers that animals might encounter in the wild, and their variable physiological and microbial effects in order to generate predictive models.

In some cases, we observed that dietary protein increased the efficiency of fiber digestion, especially for the digestion of acid detergent fiber, which represents the cellulose and lignin portion of plant materials. This outcome may be due to the fact that hindgut microbes are nitrogen limited (Reese et al., 2018), and providing additional nutrients allows for more effective fermentation or microbial digestion. Microbes can also exhibit preferences towards specific substrates, which can then yield nutritional dominance over the structure of the entire community (Estrela et al., 2021). Indeed, we observed complex interactions between diet and alterations to the microbial communities of these same rodents (Barts et al., 2025). In the future, we will work to further integrate interactions between the microbiome and aspects of host physiology measured within this experiment.

Across the species in this study, we observed an assortment of anatomical increases in size in response to diet composition. Increases in gut size can be induced through a combination of increases in cell size (hypertrophy) and cell number (hyperplasia). Intestinal atrophy and hypertrophy are observed upon refeeding in fasted pythons (Lignot et al., 2005) and on exiting hibernation in squirrels (Carey and Cooke, 1991). Similarly, diet can induce extensions in villi to increase the surface area for digestion and absorption, as has been observed in rats (Jacobs, 1983), rabbits (Chiou et al., 1994) and geese (Chiou et al., 1996). Our histological measurements did not find any evidence for hypertrophy or adjustments at the cellular level in any species, suggesting that the morphological adjustments we did observe were due to increases in cell number (hyperplasia), similar to previous studies in rats (Jacobs, 1983).

Intestinal hyperplasia for the purposes of intestinal flexibility (as opposed to an inflammatory response) is due to regulatory signals induced through epidermal growth factors and insulin-like growth factors (Steeb et al., 1994). Jejunal bypass operations or cecal ligature studies have shown localized changes in the structure of gut tissue, demonstrating that local contact between mucosa and digesta is required for some of these morphological changes (Carey and Cooke, 1991). In the case of fiber, microbial metabolites, such as short chain fatty acids produced from fermentation, can act as local nutrient sources for intestinal epithelial cells (Donohoe et al., 2011) and can induce changes in gene expression through interactions with histone deacetylase enzymes (Davie, 2003). Interestingly though, the purely physical nature of the diet, such as particle size (Munn et al., 2009), can yield lengthening or thickening of gut morphology. A biomedical study in mice demonstrated that physical stretching of the gut increases the expression of IGF-1 in these tissues, eventually leading to a doubling of intestinal length (Dunn et al., 2007), suggesting that increases could solely be due to the increased bulk of fiber material moving through the gastrointestinal tract. Overall, more work is required to understand the variety of factors and mechanisms underlying gut flexibility.

More consistent effects of diet were seen on the mass of some peripheral organs, where all three rodent species exhibited greater pancreas mass on high-protein diets and greater kidney mass when consuming high-fiber diets. Here though, there are fewer comparisons to make among our data and previous studies, as those investigating the effects of diet on digestive physiology did not measure the mass of peripheral organs (Bozinovic et al., 1997; Hume et al., 1993; Pei et al., 2001; van Zyl et al., 1999). Another feeding trial in herbivorous degus (*O. degus*) showed greater liver and kidney mass when degus were fed a higher protein diet, which may be related to the excretion of nitrogenous compounds from amino acid metabolism (Sabat and Bozinovic, 2008). In our study, we did not observe any differences in liver size, and only the grasshopper mice exhibited higher kidney mass on high-protein diets. Interestingly, across several species of prickleback fish, the liver tissues showed stronger species-specific transcriptional responses to diet, suggesting that this organ may be particularly important in allowing dietary specialization (Herrera et al., 2022). Thus, we may need to address other biological levels of physiological function and integration to understand responses of these peripheral organs.

Across several gut regions, we observed that dietary fiber induced a slightly higher pH of the luminal contents. It should be noted that the pH scale is logarithmic, and so the increase of 1.7 pH units in the foregut of the montane voles represents a 98% reduction in the concentration of hydrogen ions, which is likely to have numerous biochemical effects. Increased gastrointestinal pH in response to dietary fiber has been observed in cattle (Russell et al., 1981) and other rodent species (Kohl et al., 2013), though no fiber-induced changes to gut pH were observed in herbivorous degus (Bozinovic et al., 1997). While fiber may induce changes through higher ion binding capacity (Eastwood, 1992), or by microbial production of short-chain fatty acids (Ilhan et al., 2017), it is more likely that animals physiologically regulate the pH of the luminal space. Indeed, dietary fiber increases bicarbonate secretion from the pancreas of several species (Sommer and Kasper, 1984; Zebrowska and Low, 1987), a process that requires energy through active transport (Schulz, 1980). The changes in luminal pH could yield downstream physiological effects, as changes in pH can cause differential ionization of nutrients, enzymes, transporters and secondary chemicals (Kararli, 1995), or could induce changes in the gut microbiome (Ilhan et al., 2017). Thus, this change in the physicochemical environment may be involved in the integration of functionality across levels of organization.

We observed changes in the activity of several digestive enzymes in response to diet. In general, such shifts in activity are thought to follow the adaptive modulation hypothesis, such that the activities of enzymes match levels of dietary substrates to maintain optimal digestion while optimizing biosynthetic energy and membrane space (Karasov and Diamond, 1988; Karasov and Douglas, 2013). Indeed, we showed that pancreatic trypsin activity was higher in white-footed mice consuming high-protein diets. Similarly, the activity of intestinal maltase correlated with soluble carbohydrate content of experimental diets, as has been previously shown in white-footed mice (Wang et al., 2019). However, we did not observe protein-induced increases in the activity of the intestinal protease aminopeptidase-N in any of our rodent species. Such inductions have been demonstrated in white-footed mice (Wang et al., 2019) and laboratory mice (Raul et al., 1987), though these studies used protein differentials of >40% between groups, compared with a difference of only 13% protein in our study. Additionally, non-relevant nutrients can impact enzyme activity, such as dietary lipids decreasing the activity of intestinal aminopeptidase-N in white-footed mice (Wang et al., 2019). While lipid content was held consistent in our diets, the complex interactions across nutrients and flexibility remain to be elucidated (Karasov and Douglas, 2013).

### Phenotypic flexibility across species

When comparing flexibility across our species, we saw differential responses and integration across biological levels. PCA revealed strong and significant effects of fiber, but the magnitudes of these effects varied across species. Additionally, PCA exhibited significant interactions between species and dietary protein, demonstrating that these species mount distinct responses to dietary protein across their digestive physiology.

Herbivorous voles exhibited some elements of flexibility, often in response to fiber, though with a lower magnitude compared with white-footed mice. The reduced capacity for flexibility in montane voles compared with white-footed mice may be because the gastrointestinal system of voles has evolved a greater size to regularly cope with high-fiber diets, similar to the limited flexibility observed in herbivorous degus (Bozinovic, 1995; Bozinovic et al., 1997; Sabat and Bozinovic, 2008). In our experiment, montane voles did not exhibit increases in the efficiency of fiber digestion, but instead showed decreases in the efficiency of fiber digestion when consuming high-fiber diets. These contrasting results may be due to more nuanced digestive strategies used to digest recalcitrant materials, namely the spectrum of ‘rate maximizing’ to ‘yield maximizing’ (German et al., 2015). Digestive responses may be that of rate maximizing, by which the capacity of the digestive system functions to maximize ingestion or digestion rates through increases in food intake (German et al., 2015). In contrast, yield maximizing is associated with modifications to maintain or increase the total efficiency of digestion, including increases in gut size to retain food material and increase digestion. Fiber did not induce as drastic of phenotypic changes in montane voles as in other rodent species, and thus the digestive system of herbivorous voles may be enlarged rather constitutively given their naturally herbivorous diet. In the face of high-fiber diets, voles might not respond by increasing the efficiency of fiber digestion, but may instead rely more on digesting the easily digestible components of plant material to thrive on a herbivorous diet (Bozinovic, 1995) and conserving energy through low metabolic rates (Bozinovic et al., 2007).

In white-footed mice, our results demonstrate acclimatization of the digestive system, likely serving to increase the capacity to digest fiber and thus maximize the yield of digestion. Consistent with previous hypotheses (Buddington et al., 1991; Karasov et al., 2011), the omnivorous white-footed mouse also exhibited the greatest flexibility. Studies have demonstrated that the dimensions and mass of gastrointestinal tissues vary seasonally in wild *Peromyscus*, likely as a result of changing availability of insect prey and increased reliance on plant material during winter months (Chapman and McLean, 2023; Derting and Hornung, 2003). However, rather than a dietary response, the increased size of gastrointestinal organs could be a response to temperature and increased energy demands during colder winter months (Hammond and Kristan, 2000). Further, previous studies have linked environmental variability, including seasonal variation in weather, with increased flexibility in digestive physiology (Maldonado et al., 2011; Naya et al., 2008). While our study isolates diet composition as a source of flexibility, the full proximate and ultimate factors that determine gastrointestinal flexibility remain to be clarified.

Grasshopper mice exhibited reduced flexibility, perhaps due to some physiological canalization towards their typically carnivorous diet of arthropods and occasionally other rodents. Regardless, they still did exhibit some elements of flexibility in response to dietary fiber, such as heavier stomachs and longer and wider large intestines. Notably though, grasshopper mice did not exhibit significant increases in the mass of their cecum, which drastically increased in size in the other species. Despite this lowered flexibility, this species maintained similar digestibility levels to those of the white-footed mice, suggesting that at a functional level they are able to achieve similar digestive capacities when under captive conditions.

Overall, we observed a variety of physiological responses across our three rodents, demonstrating that each has a unique set of responses towards nutritional composition. These interspecific differences in flexibility are likely due to different evolutionary interactions with typical diets. Additional studies are needed to demonstrate whether trends of our rodents are representative of their broader feeding categories (herbivores, omnivores, carnivores). We observed species differences in physiological responses, but more consistent effects at the level of whole-animal performance (for example, all animals were more efficient at digesting protein when feeding on high-protein diets). Thus, a ‘Grand Challenge of Comparative Physiology’ remains in understanding how these biological levels integrate with one another and scale to the level of organismal performance (Mykles et al., 2010).

## Supplementary Material

10.1242/jexbio.249797_sup1 Supplementary information

Dataset 1. This file contains additional information related to this experiment.
Sheet 1 contains additional composition and nutritional information for experimental diets.
Sheet 2 contains raw data values collected from this feeding trial.
Sheet 3 contains data from gut histological measurements.
