# Supplementary material for: How consistent is ‘the dynamic gut’? Complex physiological responses to dietary fiber and protein across three rodent species
Source: J Exp Biol. 2025 Jun 20;228(14):jeb249797. doi: 10.1242/jeb.249797 (PMC12212646; doi:10.1242/jeb.249797)
Supplement: Supplementary information [file jexbio-228-249797-s1.pdf]

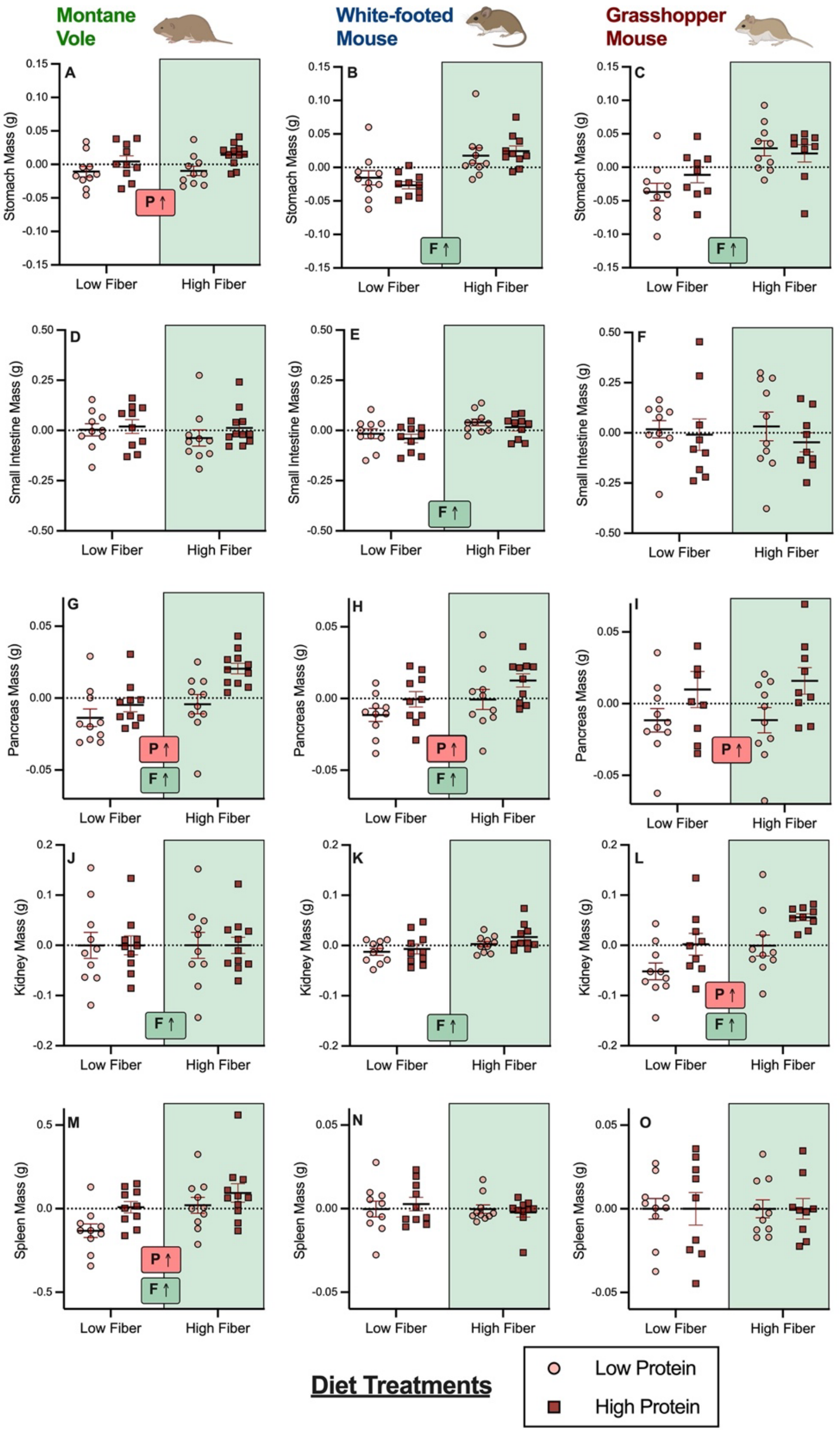

Fig. S1. Effects of diet composition on masses of the stomach, small intestine, and peripheral organs of three rodent species. Graphically, points represent residuals of organ masses from correlations with body mass. Statistically, models were run as multifactor ANCOVAs using body mass as a covariate. While residuals do not graphically depict a difference for kidney masses of voles, these results were statistically significant based on ANCOVAs. Letters and arrows inset within panels summarize significant experimental effects based on statistical results presented in Table 4. P: Protein Effect; F: Fiber Effect; P x F: Protein x Fiber Effect. Sample sizes can be found based on degrees of freedom in Tables 3 and 5.

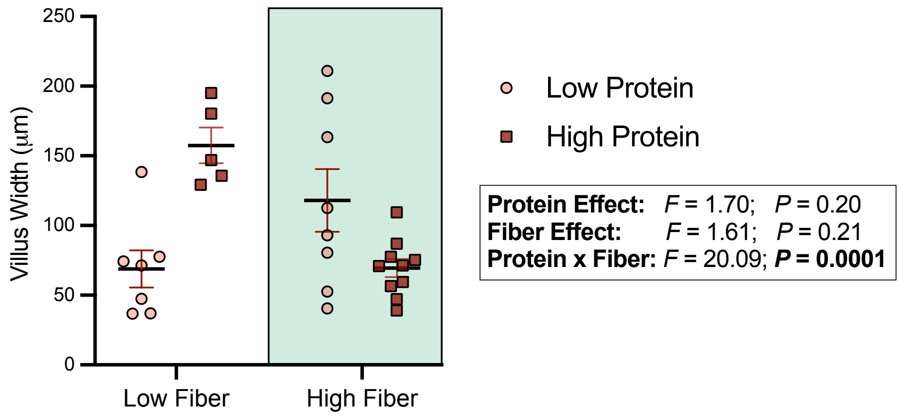

Fig. S2. Villus width of small intestines from montane voles (*Microtus montanus*) fed diets varying in fiber and protein levels. Degrees of freedom (model, error) are (3,20).

Dataset 1. This file contains additional information related to this experiment.

Sheet 1 contains additional composition and nutritional information for experimental diets.

Sheet 2 contains raw data values collected from this feeding trial.

Sheet 3 contains data from gut histological measurements.

Available for download at
https://journals.biologists.com/jeb/article-lookup/doi/10.1242/jeb.249797#supplementary-data

Journal of Experimental Biology • Supplementary information

Table S1. Statistical results of ANCOVAs for masses of stomach, small intestine, and peripheral organs, using body mass as a covariate. Degrees of freedom (model, error) for each species are as follows: voles (4,36); white-footed mice (4,35); and grasshopper mice (4,33).

	**Montane Voles**		**White-footed Mice**		**Grasshopper Mice**	
	F	*P*	*F*	*P*	*F*	*P*
**Stomach Mass**						
Fiber	0.56	0.46	22.27	**<0.0001**	16.62	**0.0003**
Protein	7.58	**0.009**	0.12	0.73	0.45	0.51
Fiber x Protein	0.46	0.50	1.06	0.31	1.82	0.19
Body Mass	37.21	**<0.0001**	15.89	**0.0003**	42.65	**<0.0001**
**Small Intestine Mass**						
Fiber	0.51	0.48	8.23	**0.007**	0.03	0.86
Protein	1.05	0.31	1.56	0.22	0.72	0.40
Fiber x Protein	0.29	0.59	0.01	0.98	0.17	0.68
Body Mass	50.53	**<0.0001**	14.37	**0.0006**	8.34	**0.007**
**Pancreas Mass**						
Fiber	9.86	**0.003**	4.58	**0.04**	0.08	0.78
Protein	9.92	**0.003**	4.56	**0.04**	6.11	**0.019**
Fiber x Protein	2.25	0.14	0.04	0.83	0.09	0.76
Body Mass	19.60	**<0.0001**	0.67	0.42	25.89	**<0.0001**
**Liver Mass**						
Fiber	3.28	0.08	0.05	0.82	0.36	0.55
Protein	1.57	0.22	0.01	0.96	2.22	0.15
Fiber x Protein	1.21	0.28	0.06	0.81	0.02	0.89
Body Mass	54.58	**<0.0001**	30.41	**<0.0001**	38.65	**<0.0001**
**Kidney Mass**						
Fiber	5.41	**0.026**	6.32	**0.017**	8.98	**0.005**
Protein	2.02	0.16	1.48	0.23	9.33	**0.004**
Fiber x Protein	0.26	0.61	0.29	0.59	0.01	0.94
Body Mass	41.74	**<0.0001**	30.00	**<0.0001**	74.17	**<0.0001**
**Spleen Mass**						
Fiber	6.51	**0.015**	0.50	0.48	1.86	0.18
Protein	5.36	**0.026**	0.03	0.87	0.28	0.60
Fiber x Protein	0.49	0.49	0.47	0.50	0.19	0.66
Body Mass	42.62	**<0.0001**	16.55	**0.0003**	9.96	**0.003**

Journal of Experimental Biology • Supplementary information
